# Exploration of the Effects of Cadmium Stress on Photosynthesis in *Oenanthe javanica* (Blume) DC.

**DOI:** 10.3390/toxics12050307

**Published:** 2024-04-23

**Authors:** Ronghua Zhou, Jun Xu, Liangjun Li, Yulai Yin, Bowen Xue, Jingjing Li, Fangfang Sun

**Affiliations:** 1 Suzhou Academy of Agricultural Sciences, Institute of Agricultural Sciences in Taihu Lake Region of Jiangsu, Suzhou 215105, China; 20220112@jaas.ac.cn (R.Z.); 20083003@jaas.ac.cn (J.X.); 20220118@jaas.ac.cn (B.X.); 20230068@jaas.ac.cn (J.L.); 2 School of Horticulture and Plant Protection, Yangzhou University, Yangzhou 225009, China; ljli@yzu.edu.cn

**Keywords:** aquatic vegetable, cadmium stress, chlorophyll fluorescence, environmental pollutants, JIP-test, photosynthetic electron transport chain, water dropwort

## Abstract

Cadmium ion (Cd^2+^) stress is a major abiotic stressor affecting plant photosynthesis. However, the impact of sustained high-concentration Cd stress on the photosynthetic electron transport chain of aquatic plants is currently unclear. Here, prompt fluorescence (PF), delayed fluorescence (DF), and P700 signals were simultaneously measured to investigate the effect of Cd stress on photosynthesis in water dropwort [*Oenanthe javanica* (Blume) DC.]. We aimed to elucidate how Cd stress continuously affects the electron transport chain in this species. The PF analysis showed that with prolonged Cd stress, the F_J_, F_I_ and F_P_ steadily decreased, accompanied by a positive shift in the K-band and L-band. Moreover, JIP-test parameters, including TR_O_/ABS, ABS/CS_O_, TR_O_/CS_O_ and PI_ABS_, were significantly reduced. The P700 signals showed that exposure to Cd stress hindered both the fast decrease and slow increase phases of the MR transient, ultimately resulting in a gradual reduction in both V_PSI_ and V_PSII−PSI_. The DF analysis showed a gradual decrease in the I_1_ and I_2_ values as the duration of stress from Cd increased. The above results suggested that Cd stress affected the photosynthetic electron transport in water dropwort by influencing the amount of active PSII and PSI, primarily affecting PSII RCs in the early to mid-stages and PSI reductive activity in the later stage.

## 1. Introduction

Heavy metal contamination is frequently observed as one of the abiotic stressors in agricultural production, especially in underdeveloped countries. The heavy metal content in agricultural land and irrigation water has constantly increased due to the heavy use of chemical fertilizers and the unreasonable discharge of “three wastes” from industrial facilities, seriously threatening the safety of aquatic vegetable production. Cd is the most toxic element among Category I harmful elements [1]. Easily absorbed by and accumulated in plants [2,3], Cd can produce toxic effects even at a low concentration [4,5]. It inhibits plant growth by causing yellowing and the shedding of leaves as well as reducing dry matter. In serious cases, it may cause plants to wither or even die [6,7,8].

Photosynthesis, a vital activity for plants, is a process in which plants fix CO_2_ and water using energy from sunlight, producing organic matter and finally releasing oxygen. Photosynthesis in plants is hindered by heavy metal stress [9,10,11,12]. Specifically, the synthesis of chlorophyll and carotene is inhibited, and the activity of key enzymes in photosynthesis is affected [13]. Furthermore, light-harvesting chlorophyll *a*/*b* binding proteins are downregulated [14], which hinders the process of photosynthesis and inhibits plant development [15,16]. Heavy metals such as Cd, Mn and Pb can markedly reduce the net photosynthetic rate (Pn), transpiration rate (E), stomatal conductance (Gs) and intercellular carbon dioxide concentration (Ci) of leaves [16,17], and result in a decrease in maximum photochemical efficiency (Fv/Fm), potential activity (Fv/Fo), PSII actual photochemical efficiency (ΦPSII), and photochemical quenching coefficient (qP) [18]. Chlorophyll fluorescence measurement technologies have recently been widely applied in the field of photosynthesis research. These technologies can be used to collect photosynthesis-related information from experimental materials without causing damage. The prompt chlorophyll *a* fluorescence (PF) transient (OJIP) can reflect the photochemical changes in photosystem II (PSII) before the activation of the dark reaction [19,20,21,22]. The modulated 820 nm reflection (MR) is an effective way to study the primary photochemical reaction and examine the redox activity of PSI [19,23]. Furthermore, the delayed chlorophyll *a* fluorescence (DF) can intuitively reflect the state of PSII. The above technologies have been extensively employed in research on stress resistance in corn, rice, wheat, and other major crops [24,25,26,27]. Cu stress reduces the number of oxygen-evolving complexes (OECs) and inhibits the photosynthetic electron transport of PSII in higher plants [28]. Barley and oilseed rape show similar responses under Cd stress [29,30]. Mn stress inhibits PSI oxidization-reduction reactions in *Melia azedarach* but not in *Ligustrum lucidum* [31]. The above studies mainly focused on xerophytic crops [32,33]. However, heavy metal ion pollution is more harmful to aquatic crops than to xerophytes due to the high mobility of these pollutants. As far as we know, there have been limited investigations into alterations in the photosynthetic function of aquatic vegetables caused by exposure to heavy metals. Furthermore, there is currently no research revealing the impact of heavy metal stress on the photosynthetic electron transport chain and its components in aquatic vegetables.

Water dropwort [*Oenanthe javanica* (Blume) DC.], a common aquatic vegetable in China, is favored by consumers because of its much higher nutritional and medicinal value compared with other common vegetables. In China, Cd pollution seriously threatens the production safety of water dropwort and other aquatic vegetables. However, there are few relevant reports about aquatic vegetables vulnerable to heavy metal pollution. Herein, the local variety “Yuqihongqin” was utilized as the experimental material to investigate the effects of high-concentration Cd stress on the photosynthetic electron transport chain and its components in water dropwort. We hypothesized that cadmium stress might impair multiple sites of the photosynthetic electron transport chain in water dropwort. Additionally, the injured sites may vary across distinct stages of Cd treatment. Our objectives were to investigate the impact of high concentration Cd stress on the photosynthetic electron transport chain and its components in water dropwort during different Cd treatment periods, to explore the target sites through which Cd^2+^ acts on photosynthetic components of water dropwort at different Cd treatment duration.

## 2. Materials and Methods

### 2.1. Plant Material and Growth Conditions

The local variety of *O. javanica*, “Yuqihongqin”, was employed as the experimental material. Planted in April 2020, the seedlings originated from the Vegetable Research Institute of Suzhou Academy of Agricultural Sciences (SAAS) and were cultivated in the Suzhou Cao-hu Agricultural Demonstration Garden. In early October 2020, seedlings with similar growth trends were randomly selected and transplanted in a vegetable experiment greenhouse for hydroponic experiments. During the experiment, the greenhouse was maintained at a temperature of 30/25 °C, with a diurnal cycle of 12/12 h, an approximate light intensity of 400 μmol m^−2^ s^−1^, and a relative humidity of 70%. After being washed with clean water, all seedlings were randomly planted in eighteen turnover boxes (43.3 cm × 30.8 cm × 14.5 cm) containing 10 L of Hoagland solution. Each box contained 10 seedlings, with a spacing of 8 cm between seedlings and a row spacing of 10 cm. During the experimental period, the nutrient solution in all the turnover boxes was supplemented every 3 d to reach a water volume of 10 L.

### 2.2. Cd Treatment

The treatment solutions were prepared with Cd chloride hemi (pentahydrate) (Sinopharm Chemical Reagent Co., Ltd., Shanghai, China: CdCl_2_∙2.5 H_2_O, F.W. 228.36; GB/T 1285-1994) to provide Cd^2+^. In this study, two treatments were established based on the preexperiment, with varying levels of Cd^2+^: (1) 0 mg L^−1^ Cd^2+^ (Cd_0_) and (2) 100 mg L^−1^ Cd^2+^ (Cd_100_). Each group included nine turnover boxes, for a total of 90 seedlings. The OJIP transient, MR transient, and DF induction decay curves of functional leaves of the water dropwort plant were measured at 0, 3, 6, 9, 12, and 15 days into Cd_0_ group or Cd_100_ group. After each fluorescence signal measurement, the plants were used to determine the pigment, hydrogen peroxide (H_2_O_2_), malondialdehyde (MDA) and Cd contents of water dropwort.

### 2.3. Determination of PF, MR, and DF

The simultaneous determination of OJIP transient, MR transient, and DF induction decay curves was performed with a multifunctional plant efficiency analyzer (M-PEA, Hansatech, Norfoik, UK) [34,35]. First, 5000 μmol m^−2^ s^−1^ red light (with a wavelength of 627 nm) was used to determine the three measures simultaneously, which was followed by light–dark conversion after exposure for 300 μs. The DF signal in the dark condition and the PF signal and MR signal in the light condition were recorded. The JIP-test of the PF curve was then carried out as described [19]. A total of sixteen parameters were developed from OJIP transients using the JIP-test (Table 1).

### 2.4. Quantification of Pigment, H_2_O_2_, MDA and Cd Contents

To determine the pigment content, 0.5 g of functional leaves were sliced into smaller pieces. Then, 10 mL of 80% acetone was added, and the mixture was stored in a freezer at −20 °C for 24 h to extract the pigments. Three milliliters of chlorophyll extract was taken, and the optical density (OD) values of the extract at 662, 645 and 470 nm were determined using a UV–Vis spectrophotometer (TU-1810, Beijing General, Beijing, China). Equations (1)–(3) were utilized to compute the quantities of chlorophyll *a*, chlorophyll *b*, and carotenoids [36].

Chlorophyll *a* = 12.25 × OD_662_ − 2.79 × OD_645_ (1)

Chlorophyll *b* = 21.50 × OD_645_ − 5.10 × OD_662_ (2)

Total carotenoids = (1000 × OD_470_ − 1.82 × Chl *a* − 85.02 × Chl *b*)/198 (3)

To determine the concentration of H_2_O_2_, 5 g of functional leaves was added to acetone (5 mL, 4 °C), ground into a homogeneous slurry, and then centrifuged at 3000 rpm for 10 min. One milliliter of the supernatant was taken and mixed with 5% titanium sulfate and concentrated ammonia, retaining the precipitate and discarding the supernatant. This process was repeated three times. The washed precipitate was completely dissolved in 5 mL of 2 mol sulfuric acid. The absorbance was measured at 415 nm using a UV–Vis spectrophotometer (TU-1810, Beijing General, Beijing, China). The H_2_O_2_ content was calculated by using Equation (4) [37].

H_2_O_2_ content = (C × Vt)/(Vs × W) (4)

where C is the concentration of H_2_O_2_ in the sample obtained by examining the standard curve; Vt is the total volume of the sample extraction solution; Vs is the volume of the sample extraction solution used during measurement; and W is the fresh weight of the sample.

The determination of malondialdehyde (MDA) content was performed via thiobarbituric acid spectrophotometry [38]. Functional leaves weighing 0.05 g were sliced into fragments before being mixed with 2 mL of a 10% TCA solution to create a paste through grinding. Next, 3 mL of 10% TCA solution was added to continue the grinding process. The mixture obtained after crushing was transferred to a centrifuge tube with a capacity of 5 mL and subsequently centrifuged at 3000 rpm for 10 min. To perform the experiment, 2 mL of the supernatant was combined with 2 mL of TBA solution and thoroughly mixed, and the mixture was heated in a water bath for 20 min. In the control, the extract was substituted with 2 mL of distilled water. Following a quick cooling process, the mixture was separated using centrifugation, and the supernatant was collected for absorbance measurements at wavelengths of 532 nm, 600 nm, and 450 nm. The MDA content was calculated by using Equation (5).

MDA content = [6.45 × (A_532_ − A_600_) − 0.56 × A_450_] × Vt/(Vs × W) (5)

where A is the absorbance value; Vt is the total volume of the sample extraction solution; Vs is the volume of the sample extraction solution used during measurement; and W is the fresh weight of the sample.

Water dropwort samples were analyzed for Cd content using microwave digestion-inductively coupled plasma–mass spectrometry (ICP–MS) [39]. Following the grinding of functional leaves of water dropwort into a fine powder, 0.2 g of the obtained sample was weighed out in a digestion jar. Then, 5 mL of HNO_3_ was introduced, and the samples were allowed to predigest at room temperature for 0.5 h. Subsequently, 2 mL of H_2_O_2_ was added. The digestion protocol was configured in the following manner: the temperature was elevated to 160 °C within a span of 10 min using 800 W and held steady for 5 min. Subsequently, the temperature was further increased to 200 °C over a duration of 10 min employing 1600 W and maintained at this level for 25 min. Following the digestion process, the sample was cooled to ambient temperature. The volume was adjusted to 50 mL using ultrapure water, and the concentration of Cd was analyzed using ICP–MS (NexION 2000 G, PerkinElmer, Waltham, MA, USA).

### 2.5. Data Processing

Both chlorophyll fluorescence and physiological parameters were statistically analyzed using SPSS 17.0 (IBM, New York, NY, USA) and plotted using SigmaPlot 12.5 (Systat Software Inc., San Jose, CA, USA). One-way analysis of variance (ANOVA) was used, and differences among different treatment durations (0, 3, 6, 9, 12, and 15 days) within the Cd_0_ group or Cd_100_ group were analyzed using Duncan’s multiple range test (*p* < 0.05). Values were expressed as the mean ± standard errors (SE) of five replicate samples (*n* = 5).

## 3. Results

### 3.1. Growth, Pigment Content, H_2_O_2_ Content, MDA Content and Cd Concentration

**Figure 1:**
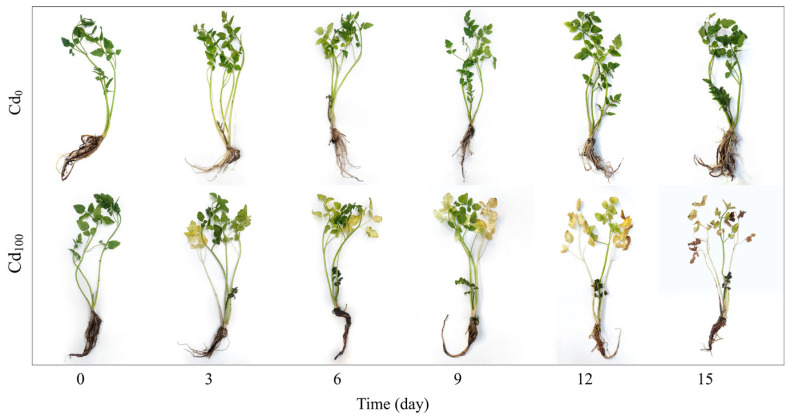
Phenotypic characterization of water dropwort in Cd_0_ and Cd_100_ groups on the 0th, 3rd, 6th, 9th, 12th and 15th days of treatment.

After 3 d of Cd_100_ treatment, the water dropwort in the Cd_100_ group showed a yellowing of leaves. As the Cd_100_ treatment continued, the plants further showed leaf wilting, stalk whitening, root rot, and plant wilting (Figure 1). The water dropwort in Cd_0_ group showed no significant changes throughout the entire experimental period.

**Figure 2:**
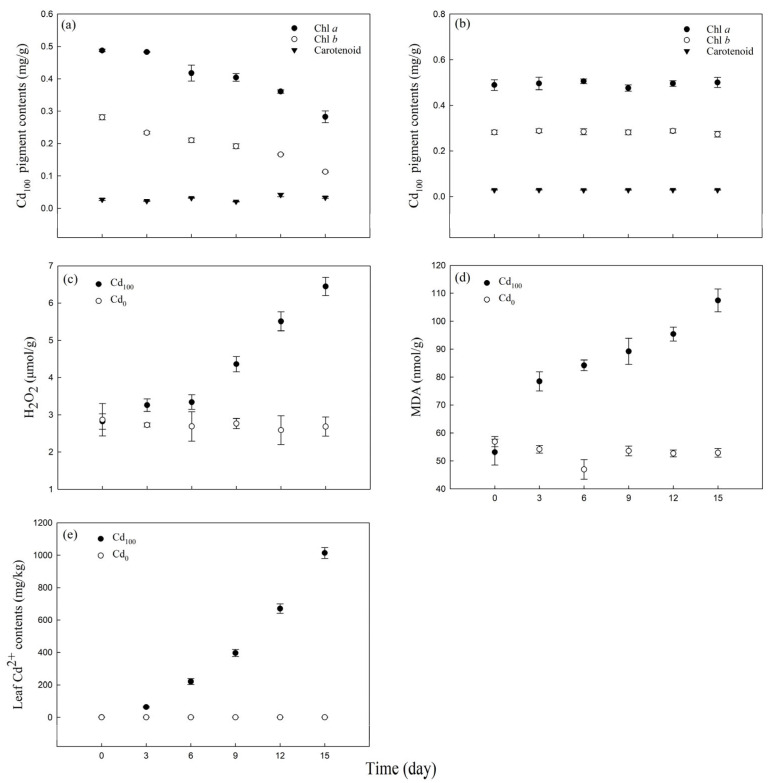
Changes in pigment (**a**,**b**), H_2_O_2_ (**c**), malondialdehyde (MDA) (**d**), and Cd^2+^ contents (**e**) in water dropwort leaves of the Cd_0_ group and the Cd_100_ group on the 0th, 3rd, 6th, 9th, 12th and 15th days of treatment (*n* = 5).

The excess Cd caused a decrease in chlorophyll *a* and chlorophyll *b* contents compared with those at 0 d but had little effect on carotenoids (Figure 2a). The chlorophyll *a* and chlorophyll *b* contents decreased by 42.02% and 59.90%, respectively, within 15 d of Cd_100_ treatment, while the carotenoid content did not change significantly (Table S1). The chlorophyll *a*, chlorophyll *b* and carotenoids contents under Cd_0_ treatment showed no significant change within 15 d of experimental period (Figure 2b).

Under Cd_0_ treatment, there was no significant change in H_2_O_2_ (Figure 2c), MDA (Figure 2d) and leaf Cd^2+^ contents (Figure 2e) within 15 d of experimental period. Under high Cd stress (Cd_100_ treatment), the H_2_O_2_ content slightly increased from 0 d to 6 d of treatment. As the treatment continued, the H_2_O_2_ content significantly increased from 6 d to 15 d of treatment (Figure 2c), rising from 3.34 μmol g^−1^ to 6.45 μmol g^−1^ (Table S1). The trend for the MDA content was also sensitive to Cd^2+^ stress (Figure 2d). The MDA content significantly increased from 53.11 nmol g^−1^ to 78.48 nmol g^−1^ after 3 d of Cd^2+^ treatment and gradually increased to 107.46 nmol g^−1^ after 15 d of Cd_100_ treatment (Table S1). Water dropwort exhibited a strong ability to absorb Cd (Figure 2e). After 3 d of Cd_100_ treatment, the leaf Cd concentration increased from 1.13 mg kg^−1^ to 64.23 mg kg^−1^. As the Cd_100_ treatment continued, the Cd concentration in the leaves increased continuously, increasing by 1478.62% (Table S1) after 15 d of Cd_100_ treatment (compared with the concentrations on the third day).

### 3.2. Effect of Cd Stress on Rapid Chlorophyll Fluorescence Kinetic Curves and JIP-Test Parameters

**Figure 3:**
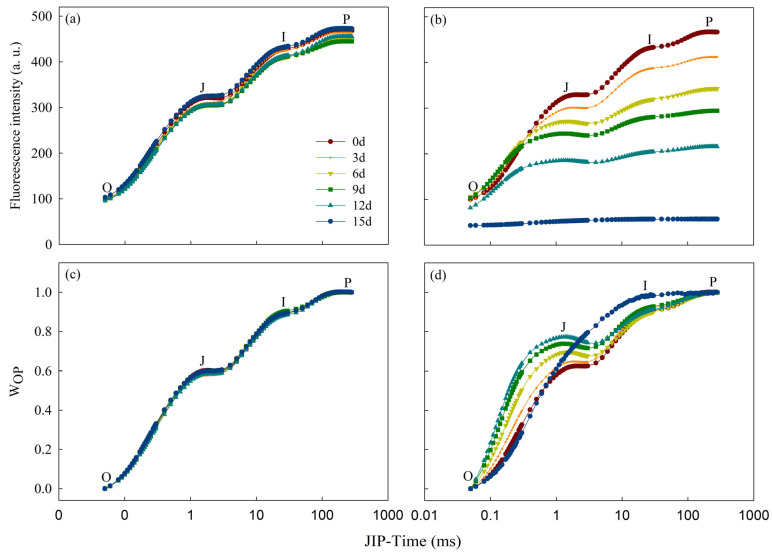
The PF transients of the Cd_0_ (**a**) group and Cd_100_ (**b**) group on the 0th, 3rd, 6th, 9th, 12th and 15th days of treatment. (**c**,**d**) O-P normalized transients of CK and TR, respectively, expressed as V_t_ = (F_t_ − F_O_)/(F_P_ − F_O_). Each curve is the average of five replicates and was plotted on a logarithmic time scale.

Throughout the entire duration of the treatment, there were no significant alterations observed in the OJIP curve of samples belonging to the Cd_0_ group (Figure 3a). Moreover, their O-P standardized curves were consistent (Figure 3c). As the Cd_100_ treatment continued, points J, I, and P on the OJIP curve gradually decreased (Figure 3b). The J-I and I-P segments almost disappeared, and weak signals were detected only in the O-J segment (K-band) after 12 d of treatment. The fluorescence signals essentially disappeared after 15 d of treatment. Based on the standardization of the O-P segment on the OJIP curve of the Cd_100_ group, point J on the standardized curve was stably elevated from Day 6 on, while point I displayed an evident rise on Day 15 (Figure 3d).

**Figure 4:**
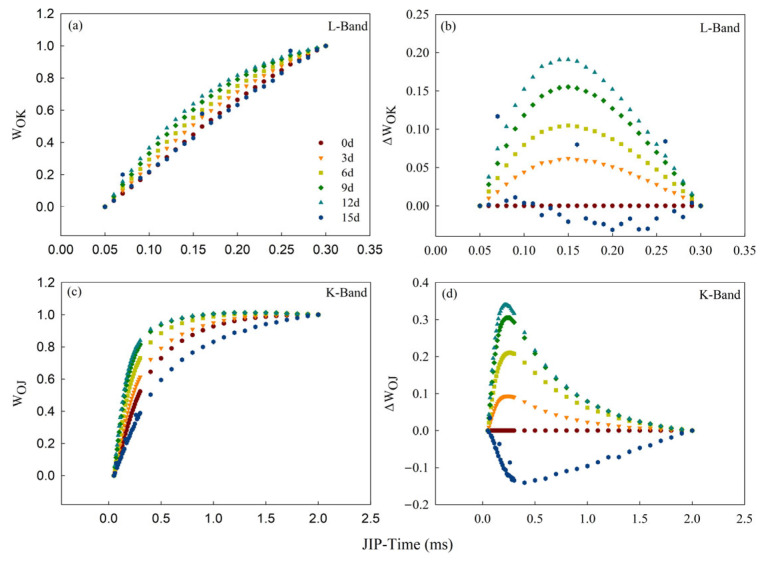
L-band and K-band of the Cd_100_ group on the 0th, 3rd, 6th, 9th, 12th and 15th days of treatment. (**a**) The normalized PF transient curves between F_O_ and F_K_, expressed as W_OK_ = (F_t_ − F_O_)/(F_K_ − F_O_), (**b**) and their variation kinetics (L-band), expressed as ΔW_OK_ = W_OK_^TR^ − W_OK_^0d^. (**c**) The PF transient curves standardized from F_O_ to F_J_, expressed as W_OJ_ = (F_t_ − F_O_)/(F_J_ − F_O_), (**d**) and their variation kinetics (K-band), represented as ΔW_OJ_ = W_OK_^TR^ − W_OJ_^0d^. Each curve is the average of five replicates.

To investigate the impact of Cd^2+^ stress on each part of the OJIP curve, the standardized O-K (L-band) curve (W_OK_) (Figure 4a) and the standardized K-band curve (W_OJ_) (Figure 4c) on the OJIP curve of the Cd_100_ group were obtained, and their differences from those on Day 0 were used to plot the difference kinetics curves ΔW_OK_ (Figure 4b) and ΔW_OJ_ (Figure 4d), respectively. A positive and rising L-band and K-band appeared after 3 d of Cd_100_ treatment (Figure 4b,d).

**Figure 5:**
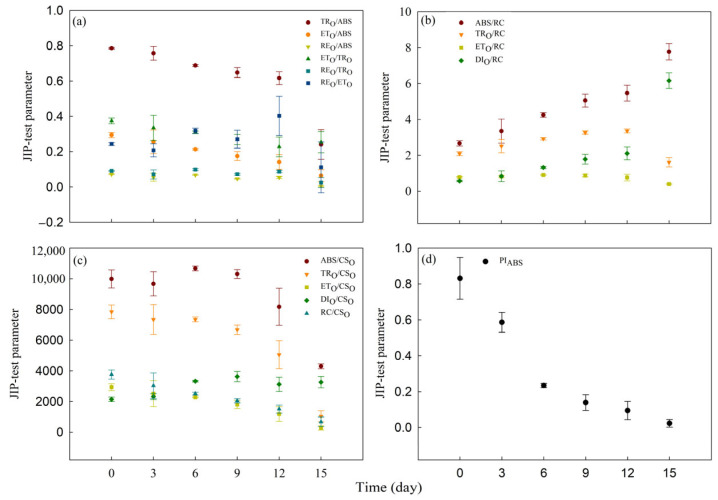
JIP-test parameters of the Cd_100_ group on the 0th, 3rd, 6th, 9th, 12th and 15th days of treatment. (**a**) TR_O_/ABS, ET_O_/ABS, RE_O_/ABS, ET_O_/TR_O_, RE_O_/TR_O_ and RE_O_/ET_O_; (**b**) ABS/RC, TR_O_/RC, ET_O_/RC, DI_O_/RC; (**c**) ABS/CS_O_, TR_O_/CS_O_, ET_O_/CS_O_, DI_O_/CS_O_, RC/CS_O_; (**d**) PI_ABS_, (*n* = 5).

To quantify alterations in photosynthetic apparatus, we developed sixteen parameters from OJIP transients using the JIP-test. Within the 15 d of Cd_0_ treatment period, the values of TR_O_/ABS, ET_O_/ABS, RE_O_/ABS, ET_O_/TR_O_, RE_O_/TR_O_, RE_O_/ET_O_, ABS/RC, TR_O_/RC, ET_O_/RC, DI_O_/RC, ABS/CS_O_, TR_O_/CS_O_, ET_O_/CS_O_, DI_O_/RC, RC/CS_O_ and PI_ABS_ of water dropwort showed no significant change (Figure S1). Within the 15 d of Cd_100_ treatment period, the values of quantum efficiencies or flux ratios parameters (TR_O_/ABS, ET_O_/ABS, RE_O_/ABS, ET_O_/TR_O_, RE_O_/TR_O_ and RE_O_/ET_O_) all significantly decreased (Figure 5a, Table S2). Additionally, ET_O_/RC, TR_O_/CS_O_, ET_O_/CS_O_, RC/CS_O_ and PI_ABS_ significantly decreased after 15 d of Cd_100_ treatment (Figure 5b,c), while ABS/RC and DI_O_/RC increased gradually with the duration of Cd_100_ treatment (Figure 5b, Table S2).

### 3.3. Effect of Cd Stress on MR

**Figure 6:**
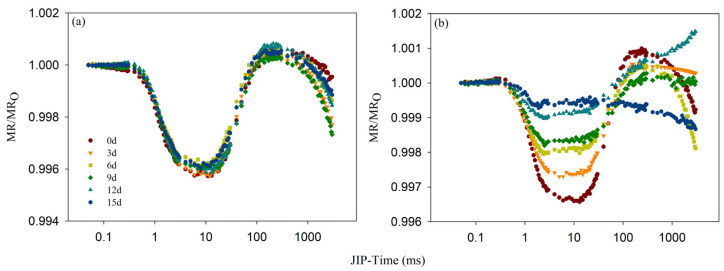
The modulated 820 nm reflection kinetics of the Cd_0_ (**a**) group and Cd_100_ (**b**) group on the 0th, 3rd, 6th, 9th, 12th and 15th days of treatment. Each curve was averaged over five replicates and plotted on a logarithmic time scale.

The redox state of PSI can be inferred from the MR curve. The MR/MR_O_ curve of the Cd_0_ group showed no significant change throughout the entire experimental period (Figure 6a). On Day 0, the MR/MR_O_ curve of the Cd_100_ group experienced two typical stages, i.e., fast-phase descent and slow-phase ascent stages (Figure 6b). After Cd_100_ treatment, both stages, as well as the lowest intersection point of the fast and slow phases, were consistently elevated. After 15 d of treatment, the ascent stage of the MR/MR_O_ curve almost disappeared, and only a small descent stage could be observed.

To intuitively observe the redox state of PSI under Cd stress, the maximum descending slope V_PSI_, the maximum ascending slope V_PSII−PSI_, and their sum V_PSII_ = V_PSI_ + V_PSII−PSI_ of the MR/MR_O_ curve were calculated. After Cd_100_ treatment, both the V_PSI_ and V_PSII−PSI_ of the TR group declined over time, cumulatively decreasing by 81.98% and 89.81% after 15 d of treatment, respectively (Table 2).

### 3.4. Effect of Cd Stress on DF

**Figure 7:**
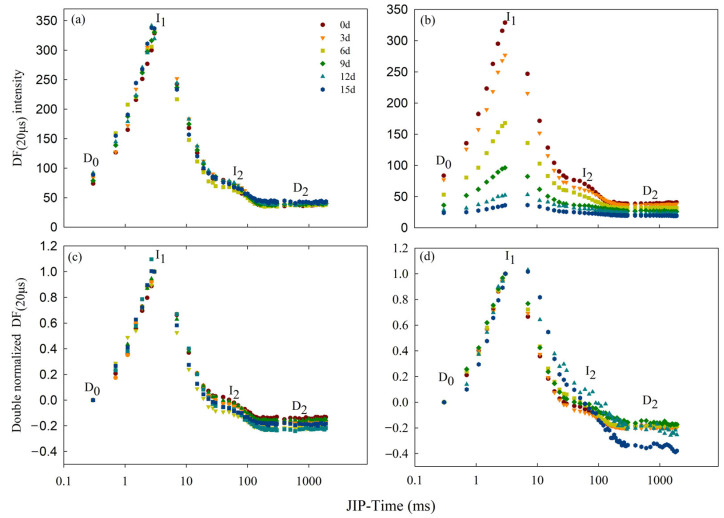
The delayed fluorescence induction kinetics of the Cd_0_ (**a**) group and Cd_100_ (**b**) group on the 0th, 3rd, 6th, 9th, 12th and 15th days of treatment. The double-normalized DF values of the Cd_0_ (**c**) group and Cd_100_ (**d**) group are expressed as (D_Ft_ − D_0_)/(DF_I1_ − D_0_). D_0_ is the initial minimum. D_2_ is the final plateau. I_1_ is the peak at 3 ms. I_2_ is the peak at 100 ms. Each curve is the average of five replicates and is plotted on a logarithmic time scale.

The DF curves at the microsecond and millisecond scales mainly exhibited the redox states of the primary electron acceptor Q_A_^−^ and the P_680_^+^ acceptor side in the photosynthetic electron transport chain. In this experiment, the DF induction curve was generated by plotting the fluorescence signals at 20 μs (Figure 7). The DF curves and the double-normalized DF values of Cd_0_ group showed no significant change throughout the entire experimental period (Figure 7a,c). After Cd_100_ treatment, the amplitudes of the DF curves of specimens in the TR group gradually declined with time, with sharp decreases at the starting point D_0_, the first peak I_1_, and the second peak I_2_ (Figure 7b). After 15 d of Cd_100_ treatment, I_1_ and I_2_ decreased by 88.95% and 58.16%, respectively. The I_2_/I_1_ value also increased significantly after Cd_100_ treatment (Table S3). After the dual standardization of D_0_ and I_1_ (Figure 7d), no obvious difference in the initial rate at which D_0_ grew to I_1_ could be observed after the treatment, while I_2_ was markedly elevated in the late Cd_100_ treatment period, consistently revealing the increased I_2_/I_1_ value in this period.

## 4. Discussion

Cd is part of a group of highly toxic heavy metals that not only affect crop quality and yield in agricultural production [8,40,41] but also affect consumer health through food chain transport [42]. Cd pollution seriously threatens the production safety of aquatic vegetables. However, there are few relevant reports about aquatic vegetables vulnerable to Cd pollution. Here, we investigated for the first time the alterations in the photosynthetic electron transport pathway induced by Cd stress in water dropwort. Furthermore, we compared the target sites of Cd stress on the photosynthetic electron transport chain under different durations of Cd_100_ treatment. We demonstrated that a short-term high concentration of Cd^2+^ treatment primarily inactivated PSII RCs, whereas with prolonged cadmium exposure, the target sites shift towards the donor side of PSI.

Under non-stress conditions, the test material “Yuqihongqin” showed no significant changes in phenotypic characterization (Figure 1), physiological parameters (Figure 2), PF (Figure 3a), MR (Figure 6a) and DF (Figure 7a) within the 15 d of hydroponic treatment. However, under Cd_100_ treatment, symptoms of wilting, leaf wilting, and stalk whitening appeared in the plants (Figure 1). This suggests that excessive levels of Cd^2+^ hinder the growth and development of water dropwort. Water dropwort cultivated hydroponically exhibited a robust absorption of Cd^2+^ (Figure 2e). The accumulation of Cd^2+^ resulted in a significant decrease in chlorophyll *a* and chlorophyll *b* content (Figure 2a, Table S1). This result confirmed the previous conclusion that Cd^2+^ has an impact on the photosynthetic process [27,43,44,45,46]. The Cd_100_ treatment also resulted in a significant increase in H_2_O_2_ (Figure 2c) and MDA (Figure 2d) contents in water dropwort. This indicates that the continuous accumulation of Cd^2+^ in leaves increases the degree of cell oxidation damage, damaging the cell membrane and leading to the dysfunction of cell membrane [47,48]. The chlorophyll *b* content was more sensitive than chlorophyll *a* to Cd stress in this study (Table S1). As most of the chlorophyll *b* is associated with the light-harvesting complex I and II, a decline would suggest degradation of the antenna. Similarly, a decrease in chlorophyll *a* content would suggest a reduction in the amount of active PSII and PSI [49,50]. A similar alteration was reported for the purslane plants’ reduction in chlorophyll *a* and chlorophyll *b* content by 35 and 41%, respectively, when 300 mg kg^−1^ of Cd was added to the soil [51]. On the contrary, chlorophyll *a* content is more sensitive to Cd stress than chlorophyll *b* in soybean plants and lettuce plants [33,52]. The above results indicated that the extent of damage to chlorophyll *a* and chlorophyll *b* varies under Cd stress among different species.

The alterations in the primary photochemical reaction of PSII are mainly indicated by the OJIP curve. In this study, point J, point I, and point P on the water dropwort OJIP curve gradually fell with increasing Cd stress duration (Figure 3b). The decrease in fluorescence intensity indicates hindered photosynthetic electron transmission. A reduction in photosynthetic pigments, along with increases in MDA and H_2_O_2_ levels in physiological indicators, serves as strong evidence for this. Membrane damage and the degradation of photosynthetic pigments affect electron transmission between the photosystems. Point J on the standardized OJIP curve rose gradually, and the J-I segment rose significantly at the late stage of Cd_100_ treatment (Figure 3d). The electron transport rate from Q_A_ to Q_B_ in the photosynthetic electron transport chain is reflected by Point J on the OJIP curve [53,54]. The increased level J in the standardized curve indicates the hindrance of electron transfer from Q_A_ to Q_B_, indicating that the rate of electron transfer at Q_A_ in the photosynthetic electron transport chain can be suppressed by Cd stress. The J-I segment on the standardized curve gradually rose after treatment, which indicates that PSII RCs were strongly repressed by Cd stress. The number of PSII RCs in water dropwort begins to decrease under the effect of Cd stress [27]. F_P_ is a complex parameter that is dependent on the structural leaf tissue characteristics and the chlorophyll content in the leaf. Lower F_P_ is related to the reduced chlorophyll *a* level (Figure 2a) [27,54,55], heightened non-radiative dissipation of PSII antenna chlorophylls [56], diminished PSII antenna size [57], impairment at the PSI accepter side [20], and/or decreased number of photosynthetic apparatus with fully closed PSII RCs [27,35]. This quasi-quenching effect on fluorescence yield indicates an inhibition of PSII electron transfer, decreasing the number of active reaction centers capable of supporting electron transfer to PSI [35,58,59,60]. The I-P segment has a relationship with the redox activity of PSI [23,61]. The segment was gradually reduced after the addition of Cd^2+^ in this study (Figure 3b), indicating that the redox activity of PSI is also affected by Cd stress. In addition, a favorable K-band and L-band appeared (Figure 4b,d). Similar phenomena have been found in other stressed crops, including corn [34], wheat [27] and rice [3]. The status of PSII components is well reflected by the L-band and K-band, which have been widely applied in research on photosynthesis under various kinds of stresses. The connectivity between all PSII components is mainly represented by the L-band [62,63], while the activity of the OEC and the electron transport ability of the donor side of PSII are represented by the K-band [61,64]. The K/L-band was positive and constantly increased with the continuous Cd_100_ treatment in this study, suggesting that Cd_100_ stress has a strong inhibitory effect on the connectivity of the OEC of PSII and among all PSII components in water dropwort.

In addition, all sixteen parameters of the JIP-test were measured to reveal the changes in each step of the photosynthetic process at different Cd_100_ treatment stages in water dropwort. JIP-test parameters allow the quantitative analysis of changes in the structure and function of components of the photosynthetic electron transport chain [58]. In the present study, all these parameters were changed by Cd stress. Most JIP test parameters exhibited a gradual change during the early to mid-stages of Cd_100_ treatment, followed by a rapid alteration at the later stage of treatment. The drop in TR_O_/ABS indicates that the light use efficiency of PSII RCs is lowered by Cd stress [39,65]. PI_ABS_, a comprehensive performance index of PSII [25,58,66], is a more sensitive response index than TR_O_/ABS in quantitatively detecting the toxicity of heavy metals [67]. In this study, PI_ABS_ was more sensitive than TR_O_/ABS to Cd stress. The drop in PI_ABS_ indicates that Cd stress strongly inhibits the PSII activity of water dropwort. The increased DI_O_/RC and DI_O_/CS_O_ suggest that Cd stress disrupted the photosynthetic electron transport process and induced the passive energy dissipation. After 12 d to 15 d of Cd_100_ treatment, there was a significant decline in RE_O_/ET_O_ and ABS/CS_O_. The decline in RE_O_/ET_O_ indicates a reduction in the transfer of electrons from Q_A_^−^ to the accepter side of PSI. The decline in ABS/CS_O_ absorbed by the antenna pigments occurred during the later stages of treatment, suggesting a substantial degradation of the antenna pigments [27]. This is consistent with the decline in chlorophyll *b* content (Figure 2a). Moreover, although chlorophyll *b* is more sensitive to Cd stress than chlorophyll *a* (Table S1), the rise in ABS/RC and the decline in RC/CS_O_ indicates that cadmium accumulation has a much greater impact on the quantity of PSII RCs than on the light-harvesting complex I and II. This aligns with the J-I standardized curve (Figure 3b) [24,25]. This indicates that the early reduction in chlorophyll *b* does not impact the absorption of light energy by the antenna. The quantities of light-harvesting complexes I and II are redundant; even with partial antenna degradation, the remaining antenna complex can still absorb sufficient light and transfer electrons downstream to the photosynthetic electron transport chain. Instead, the crucial factor influencing the efficiency of photosynthetic electron transport is the amount of active PSII and PSI in chlorophyll *a*.

The MR/MR_O_ curve indicated that Cd stress weakened the photochemical function of PSI. The descending and ascending phases of the MR/MR_O_ curve, respectively, reflect the oxidation and reduction in PSI [57]. Within 12 d after treatment, the decreases in the V_PSI_ and V_PSII − PSI_ rates were practically identical (Table 2), suggesting that the photochemical function of PSI during this timeframe is primarily constrained by the rate of electron transport from the donor side of PSII to the receptor side of PSI. The suppressive effect of Cd stress on PSII surpassed its effect on PSI. The decrease in PSI reduction activity during the early to mid-stages of Cd_100_ treatment can be attributed to the reduced number of active PSII RCs, leading to a decrease in electron supply capacity. [23,36]. This deduction is consistent with the decrease in RC/CS_O_ (Figure 5c) and the chlorophyll *a* content (Figure 2a). After 15 d of treatment, V_PSII − PSI_ showed no indication, and V_PSI_ displayed a faint indication (Table 2). This indicates that Cd stress completely eliminates the reduction activity of PSI while partially preserving its oxidation activity. The inhibitory effect of Cd stress on PSI was stronger than that on PSII at this time. The potential decline in the photosynthetic capacity of water dropwort could primarily be attributed to the vanishing reduction function of PSI [20].

The DF results demonstrated that the DF intensity of water dropwort decreased gradually due to Cd stress (Figure 7b), implying that the number of active PSII RCs could be lessened by Cd stress, weakening the ability of PSII to supply electrons for downstream processes [19,68]. This is consistent with the conclusions drawn from the decrease in chlorophyll content (Figure 2a), RC/CS_O_ (Figure 5c) and MR parameters (Table 2). Point I_1_ of the DF is correlated with the electron transport ability of the donor/receptor side of PSII and the number of PSII RCs [19,69]. Therefore, the drop in I_1_ (Figure 7a) is attributed to the reduction in the number of PSII RCs and the decreased electron transport rate from Q_A_ to Q_B_. Point I_2_ of the DF curve typically corresponds to the I-P phase on the OJIP curve and the ascending phase on the MR/MR_O_ curve, which is associated with the reduction in the PSI accepter side [26,70]. Consistent with the aforementioned results of PF and MR, the decrease in I_1_ and I_2_ (Figure 7b) indicates a reduction in the quantity of PSII RCs and decrease in the reduction activity of PSI with the continuous accumulation of Cd^2+^ [64]. Other studies [20,71,72] have noted that the I_2_/I_1_ value is associated with the electron transport ability of the PSII donor side. This value significantly increased with Cd_100_ treatment duration in this study (Table S3), indicating that the electron transport ability of the PSII donor side declined uninterruptedly, consistent with the results for the pigment changes, I-P segment, K-band, JIP-test and MR curves above.

## 5. Conclusions

This study examined the impact of exposure to elevated levels of Cd^2+^ on the photosynthetic electron transport chain and its constituents in water dropwort during different treatment periods. The findings indicated that exposure to Cd^2+^ had a detrimental effect on various components of the photosynthetic electron transport pathway in water dropwort. At the early and middle stages of Cd_100_ treatment, the damage caused by cadmium accumulation is more pronounced on PSII than PSI. Cd^2+^ stress led to the inactivation of PSII reaction centers (RCs), destruction of the oxygen evolving complex (OEC), obstruction of electron transfer from Q_A_ to Q_B_, decreased connectivity between independent PSII units, and the blockage of PSI accepter-side electron transporters in water dropwort leaves. At the later stages of Cd_100_ treatment, the numbers of active PSII RCs were significantly reduced, and the connectivity between independent PSII units further deteriorated. The disruption of the photosynthetic electron transport chain due to cadmium accumulation extended to the donor side of PSI, resulting in the loss of PSI reduction activity. At this time, the downstream pathways are blocked, although PSII still has some photosynthetic activity. In summary, the amount of active PSII and PSI associated with chlorophyll *a* is crucial in influencing the changes in the photosynthetic electron transport. A short-term high-concentration Cd^2+^ treatment primarily inactivated PSII RCs and affected the photosynthetic electron transport of PSII. With prolonged Cd^2+^ treatment, the target sites shift to the donor side of PSI and completely inhibit the reduction activity of PSI.

## Figures and Tables

**Table 1:** The derivation and meaning of technical fluorescence parameters and related JIP-test parameters.

Technical Fluorescence Parameters	Meaning
F_O_	Minimal fluorescence yield of the dark-adapted state
F_K_	PF intensity at the K-step (0.3 ms)
F_J_	PF intensity at the J-step (2 ms)
F_I_	PF intensity at the I-step (30 ms)
F_M_ = F_P_	Maximal fluorescence yield of the dark-adapted state
V_t_ = (F_t_ − F_O_)/(F_M_ − F_O_)	Relative variable fluorescence at time t
V_J_ = (F_J_ − F_O_)/(F_M_ − F_O_)	Relative variable fluorescence at the J-step (2 ms)
V_I_ = (F_I_ − F_O_)/(F_M_ − F_O_)	Relative variable fluorescence at the I-step (30 ms)
M_O_ = 4·(F_300μs_ − F_O_)/(F_M_ − F_O_)	Approximated initial slope of the fluorescence transient
Quantum efficiencies or flux ratios	
TR_O_/ABS = φ_Po_ = 1 − F_O_/F_M_	Maximum quantum yield for primary photochemistry
ET_O_/ABS = φ_Eo_ = 1 − F_J_/F_M_	Quantum yield of the electron transport flux from Q_A_ to Q_B_
RE_O_/ABS = φ_Ro_ = 1 − F_I_/F_M_	Quantum yield of the electron transport flux until the PSI electron acceptors
ET_O_/TR_O_ = ψ_Eo_ = 1 − V_J_	The efficiency of electron movement at Q_A_
RE_O_/TR_O_ = ψ_Ro_ = 1 − V_I_	Efficiency with which a PSII trapped electron is transferred until PSI acceptors
RE_O_/ET_O_ = δ_Ro_ = (1 − V_I_)/(1 − V_J_)	The efficiency of an electron beyond Q_A_ reduced PSI acceptors
Specific energy fluxes [per Q_A_-reducing PSII reaction center (RC)]	
ABS/RC = M_O_·(1/V_J_)/(1/φ_Po_)	Absorption flux per RC
TR_O_/RC = M_O_·(1/V_J_)	Trapped energy flux per RC (at t = 0)
ET_O_/RC = M_O_·(1/V_J_)·ψ_Eo_	Electron transport flux per RC (at t = 0)
DI_O_/RC = (ABS/RC) − (TR_O_/RC)	Dissipated energy flux per RC (at t = 0)
Phenomenological energy fluxes [per excited cross-section (CS)]	
ABS/CS_O_ ≈ F_O_	Absorption flux per CS (at t = 0)
TR_O_/CS_O_ = φ_Po_·(ABS/CS_O_)	Trapped energy flux per CS (at t = 0)
ET_O_/CS_O_ = φ_Eo_·(ABS/CS_O_)	Electron transport flux per CS (at t = 0)
DI_O_/CS_O_ = (ABS/CS_O_) − (TR_O_/CS_O_)	Dissipated energy flux per CS (at t = 0)
Density of reaction centers	
RC/CS_O_ = φ_Po_·(V_J_/M_O_)·(ABS/CS_O_)	Density of RCs (Q_A_-reducing PSII reaction centers)
Performance indexes	
PI_ABS_ = (RC/ABS)·[φ_Po_/(1 − φ_Po_)]·[ψ_Eo_/(1 − ψ_Eo_)]	Performance index (potential) for energy conservation from photons absorbed by PSII to the reduction of intersystem electron acceptors

**Table 2:** MR parameters of the Cd_100_ group on the 0th, 3rd, 6th, 9th, 12th and 15th days of treatment.

Treatment Duration in Days	V_PSI_	V_PSII−PSI_	V_PSII_
0 d	0.616 ± 0.097 a	0.041 ± 0.004 a	0.657 ± 0.101 a
3 d	0.470 ± 0.141 b	0.029 ± 0.012 b	0.499 ± 0.152 b
6 d	0.342 ± 0.022 c	0.021 ± 0.002 c	0.363 ± 0.023 c
9 d	0.300 ± 0.028 c	0.015 ± 0.001 d	0.315 ± 0.029 c
12 d	0.160 ± 0.081 d	0.012 ± 0.004 e	0.171 ± 0.085 d
15 d	0.111 ± 0.061 e	0.000 ± 0.003 f	0.068 ± 0.064 e

Value are means ± SD (*n* = 5). Duncan’s multiple range test is used for multiple comparisons. Lowercase letters within the same column indicate significant differences at the *p* < 0.05 level.

## Data Availability

All original contributions presented in the study are included in the article. Further inquiries can be directed to the corresponding author.

## Supplementary Materials

The following supporting information can be downloaded at: https://www.mdpi.com/article/10.3390/toxics12050307/s1, Figure S1: Relative changes in JIP-test parameters after 15 d of Cd_0_ treatment. (a) TR_O_/ABS, ET_O_/ABS, RE_O_/ABS, ET_O_/TR_O_, RE_O_/TR_O_ and RE_O_/ET_O_; (b) ABS/RC, TR_O_/RC, ET_O_/RC, DI_O_/RC; (c) ABS/CS_O_, TR_O_/CS_O_, ET_O_/CS_O_, DI_O_/CS_O_, RC/CS_O_; (d) PI_ABS_. The average of five replicates is used as the value, and the error was determined based on this value (*n* = 5).; Table S1: Physiological parameters of the Cd_100_ group on the 0th, 3rd, 6th, 9th, 12th and 15th days of treatment; Table S2: Physiological parameters of the Cd_100_ group on the 0th, 3rd, 6th, 9th, 12th and 15th days of treatment; Table S3: DF parameters of the Cd_100_ group on the 0th, 3rd, 6th, 9th, 12th and 15th days of treatment.
