# Supplementary material for: Exploration of the Effects of Cadmium Stress on Photosynthesis in Oenanthe javanica (Blume) DC
Source: Toxics. 2024 Apr 23;12(5):307. doi: 10.3390/toxics12050307 (PMC11125355; doi:10.3390/toxics12050307)
Supplement: Supplementary file 1 [file toxics-12-00307-s001.zip › Fig. S1.pdf]

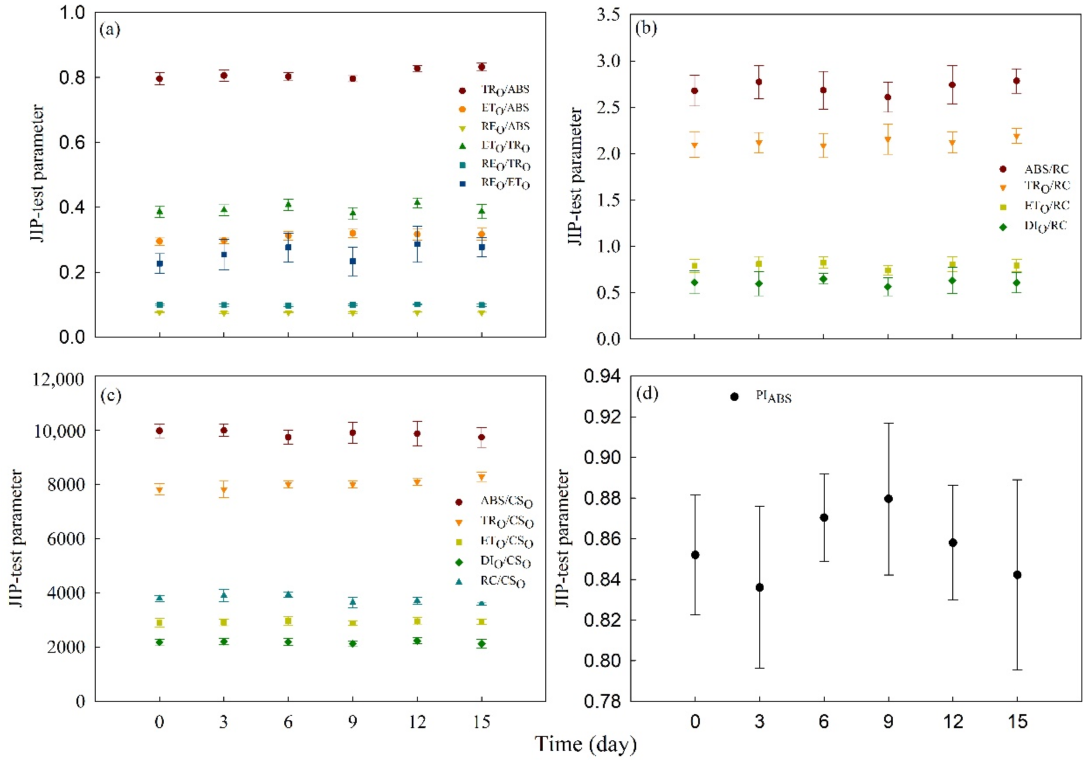

Figure S1. Relative changes in JIP-test parameters after 15 d of Cd_0 treatment. (a) TR_O/ABS, ET_O/ABS, RE_O/ABS, ET_O/TR_O, RE_O/TR_O and RE_O/ET_O; (b) ABS/RC, TR_O/RC, ET_O/RC, DI_O/RC; (c) ABS/CS_O, TR_O/CS_O, ET_O/CS_O, DI_O/CS_O, RC/CS_O; (d) PI_{ABS}. The average of five replicates is used as the value, and the error was determined based on this value (n = 5).
