# Supplementary material for: Exploration of the Effects of Cadmium Stress on Photosynthesis in Oenanthe javanica (Blume) DC
Source: Toxics. 2024 Apr 23;12(5):307. doi: 10.3390/toxics12050307 (PMC11125355; doi:10.3390/toxics12050307)
Supplement: Supplementary file 1 [file toxics-12-00307-s001.zip › Table. S1.pdf]

Table S1. Physiological parameters of the Cd_{100} group on the 0th, 3rd, 6th, 9th, 12th and 15th days of treatment.

	Chl a	Chl b	Car	H_2O_2	MDA	Cd^{2+} contents
0 d	0.488 ± 0.004 a	0.281 ± 0.008 a	0.027 ± 0.003 c	2.819 ± 0.207 e	53.106 ± 4.616 e	1.134 ± 0.493 f
3 d	0.483 ± 0.002 a	0.234 ± 0.004 b	0.023 ± 0.001 d	3.259 ± 0.170 de	78.478 ± 3.418 d	64.226 ± 5.397 e
6 d	0.418 ± 0.025 b	0.211 ± 0.007 c	0.032 ± 0.001 b	3.340 ± 0.196 d	84.201 ± 1.907 c	220.896 ± 17.392 d
9 d	0.404 ± 0.012 c	0.192 ± 0.007 d	0.021 ± 0.001 d	4.359 ± 0.205 c	89.210 ± 4.688 c	396.215 ± 22.162 c
12 d	0.361 ± 0.005 d	0.166 ± 0.002 e	0.042 ± 0.005 a	5.509 ± 0.257 b	95.396 ± 2.478 b	670.635 ± 29.353 b
15 d	0.280 ± 0.018 e	0.113 ± 0.003 f	0.033 ± 0.003 b	6.447 ± 0.243 a	107.464 ± 4.075 a	1013.947 ± 33.605 a

Value are means ± SD (n = 5). Duncan's multiple range test is used for multiple comparisons. Lowercase letters within the same column indicate significant differences at the $P < 0.05$ level.
