# Supplementary material for: Exploration of the Effects of Cadmium Stress on Photosynthesis in Oenanthe javanica (Blume) DC
Source: Toxics. 2024 Apr 23;12(5):307. doi: 10.3390/toxics12050307 (PMC11125355; doi:10.3390/toxics12050307)
Supplement: Supplementary file 1 [file toxics-12-00307-s001.zip › Table. S2.pdf]

Table S2. Physiological parameters of the Cd_{100} group on the 0th, 3rd, 6th, 9th, 12th and 15th days of treatment.

	TR_O/ABS	ET_O/ABS	RE_O/ABS	ET_O/TR_O	RE_O/TR_O	RE_O/ET_O	ABS/CS_O	TR_O/CS_O
0 d	0.786 ± 0.005 a	0.294 ± 0.014 a	0.071 ± 0.002 a	0.374 ± 0.016 a	0.091 ± 0.003 ab	0.243 ± 0.008 b	9989.833.000 ± 588.214 a	7848.839 ± 444.876 a
3 d	0.757 ± 0.039 ab	0.256 ± 0.067 ab	0.055 ± 0.022 ab	0.334 ± 0.072 ab	0.071 ± 0.026 b	0.207 ± 0.037 bc	9668.000 ± 787.370 a	7348.039 ± 970.720 bc
6 d	0.689 ± 0.006 bc	0.213 ± 0.004 bc	0.068 ± 0.003 a	0.310 ± 0.007 abc	0.099 ± 0.006 a	0.318 ± 0.015 ab	10674.000 ± 151.286 a	7349.509 ± 150.819 bc
9 d	0.648 ± 0.029 cd	0.175 ± 0.026 cd	0.046 ± 0.002 b	0.269 ± 0.029 bcd	0.072 ± 0.005 ab	0.271 ± 0.051 b	10370.330 ± 288.185 a	6681.013 ± 305.626 c
12 d	0.617 ± 0.036 d	0.141 ± 0.042 d	0.054 ± 0.006 ab	0.227 ± 0.056 d	0.087 ± 0.006 ab	0.403 ± 0.111 a	8174.500 ± 1206.701 b	5052.563 ± 906.451 d
15 d	0.240 ± 0.084 e	0.064 ± 0.034 e	0.004 ± 0.004 c	0.253 ± 0.060 cd	0.025 ± 0.028 c	0.111 ± 0.144 c	4296.667 ± 161.203 c	1033.257 ± 361.081 e
	ABS/RC	TR_O/RC	ET_O/RC	DI_O/RC	PI_{ABS}	ET_O/CS_O	DI_O/CS_O	RC/CS_O
0 d	2.664 ± 0.151 c	2.093 ± 0.110 e	0.783 ± 0.034 a	0.571 ± 0.043 e	0.831 ± 0.116 a	2938.402 ± 221.566 a	2140.994 ± 153.179 c	3759.032 ± 296.916 a
3 d	3.315 ± 0.669 bc	2.512 ± 0.374 d	0.818 ± 0.057 a	0.833 ± 0.295 de	0.586 ± 0.055 b	2514.700 ± 848.695 ab	2319.961 ± 197.298 c	3027.672 ± 830.412 ab
6 d	4.248 ± 0.132 bc	2.924 ± 0.065 c	0.905 ± 0.032 a	1.324 ± 0.067 cd	0.234 ± 0.012 c	2274.826 ± 27.954 ab	3324.491 ± 56.892 ab	2514.927 ± 96.223 bc
9 d	5.052 ± 0.362 bc	3.268 ± 0.091 ab	0.877 ± 0.076 a	1.783 ± 0.272 bc	0.139 ± 0.044 cd	1801.994 ± 266.546 bc	3626.320 ± 328.930 a	2046.511 ± 136.848 cd
12 d	5.464 ± 0.442 ab	3.359 ± 0.114 a	0.763 ± 0.187 a	2.106 ± 0.355 b	0.094 ± 0.051 d	1181.409 ± 477.941 c	3121.937 ± 465.650 b	1503.533 ± 265.565 d
15 d	7.769 ± 0.457 a	1.612 ± 0.258 d	0.398 ± 0.033 b	6.157 ± 0.432 a	0.023 ± 0.021 d	272.095 ± 138.730 d	3263.410 ± 377.499 ab	673.318 ± 308.575 e

Value are means ± SD (n = 5). Duncan's multiple range test is used for multiple comparisons. Lowercase letters within the same column indicate significant differences at the $P < 0.05$ level.
