# Supplementary material for: Exploration of the Effects of Cadmium Stress on Photosynthesis in Oenanthe javanica (Blume) DC
Source: Toxics. 2024 Apr 23;12(5):307. doi: 10.3390/toxics12050307 (PMC11125355; doi:10.3390/toxics12050307)
Supplement: Supplementary file 1 [file toxics-12-00307-s001.zip › Table. S3.pdf]

Table S3. DF parameters of the Cd_{100} group on the 0th, 3rd, 6th, 9th, 12th and 15th days of treatment.

	I_1	I_2	I_2/I_1
0 d	328.78 ± 12.79 a	56.96 ± 3.43 a	0.174 ± 0.009 a
3 d	276.94 ± 8.35 b	53.73 ± 3.24 b	0.196 ± 0.006 b
6 d	167.96 ± 6.28 c	42.40 ± 2.57 c	0.254 ± 0.015 c
9 d	96.29 ± 5.64 d	33.53 ± 2.62 d	0.347 ± 0.018 d
12 d	52.34 ± 2.17 e	28.06 ± 1.68 e	0.537 ± 0.017 e
15 d	36.35 ± 2.35 f	22.82 ± 2.49 f	0.805 ± 0.033 f

Value are means ± SD (n = 5). Duncan's multiple range test is used for multiple comparisons. Lowercase letters within the same column indicate significant differences at the $P < 0.05$ level.
